# Characterizing the allele-specific gene expression landscape in high hyperdiploid acute lymphoblastic leukemia with BASE

**DOI:** 10.1038/s41598-024-73743-8

**Published:** 2024-10-05

**Authors:** Jonas Andersson, Efe Aydın, Rebeqa Gunnarsson, Henrik Lilljebjörn, Thoas Fioretos, Bertil Johansson, Kajsa Paulsson, Minjun Yang

**Affiliations:** 1 Department of Laboratory Medicine, Division of Clinical Genetics, Lund University, Lund, Sweden; 2 Lund University Diabetes Centre, Department of Clinical Sciences Malmö, Lund University, Malmö, Sweden; 3 Department of Clinical Genetics, Pathology, and Molecular Diagnostics, Office for Medical Services, Laboratory Medicine, Region Skåne, Lund, Sweden

**Keywords:** Data processing, Software, Leukaemia, Dosage compensation

## Abstract

Somatic copy number variations (CNVs), including abnormal chromosome numbers and structural changes leading to gain or loss of genetic material, play a crucial role in initiation and progression of cancer. CNVs are believed to cause gene dosage imbalances and modify cis-regulatory elements, leading to allelic expression imbalances in genes that influence cell division and thereby contribute to cancer development. However, the impact of CNVs on allelic gene expression in cancer remains unclear. Allele-specific expression (ASE) analysis, a potent method for investigating genome-wide allelic imbalance profiles in tumors, assesses the relative expression of two alleles using high-throughput sequencing data. However, many existing methods for gene-level ASE detection rely on only RNA sequencing data, which present challenges in interpreting the genetic mechanisms underlying ASE in cancer. To address this issue, we developed a robust framework that integrates allele-specific copy number calls into ASE calling algorithms by leveraging paired genome and transcriptome data from the same sample. This integration enhances the interpretability of the genetic mechanisms driving ASE, thereby facilitating the identification of driver events triggered by CNVs in cancer. In this study, we utilized BASE to conduct a comprehensive analysis of ASE in high hyperdiploid acute lymphoblastic leukemia (HeH ALL), a prevalent childhood malignancy characterized by gains of chromosomes X, 4, 6, 10, 14, 17, 18, and 21. Our analysis unveiled the comprehensive ASE landscape in HeH ALL. Through a multi-perspective examination of HeH ASEs, we offer a systematic understanding of how CNVs impact ASE in HeH, providing valuable insights to guide ASE studies in cancer.

## Introduction

In diploid organisms, the maternal and paternal gene alleles are generally expressed equally. However, there is a group of genes in the genome where transcription originates predominantly from one allele, a phenomenon known as allele-specific expression (ASE). In humans, ASE is often associated with epigenetic inactivation in the X-chromosome or autosomal imprinting, an epigenetic phenomenon resulting in differential parent-origin gene expression^1^. ASE may also be driven by genetic variation. For example, ASE can occur due to genetic variants that lead to alternative splicing or cis-regulatory mutations that cause the two alleles to be activated by different upstream regulators. Additionally, ASE analysis of somatic mutations has unveiled a preferential selection for the expression of some alleles with well-known driver mutations, contributing to the clonal outgrowth of tumors^2^. In tumor cells, an important class of events is somatic copy number variation (CNV). The pathogenetic impact of CNVs is believed to be caused by gene dosage imbalances and allelic expression imbalance of genes that affect cell proliferation. For instance, the partial tandem duplication of the *KMT2A* gene is linked to upregulated *KMT2A* expression in acute myeloid leukemia (AML), contributing to AML development by promoting uncontrolled cell proliferation and inhibiting normal cellular differentiation^3^. Aneuploidy, *i.e.*, an abnormal number of chromosomes, is commonly observed in many cancer cells^4^. Evidence from RNA sequencing (RNA-seq) of aneuploid cells suggests upregulation of trisomic genes (those localized on trisomic chromosomes) at the mRNA level when compared to their disomic counterparts^4,5^. However, the extent to which aneuploidy alters the allelic expression of genes in cancer and the overall role of ASE in CNVs remain unknown. ASE analysis generally involves counting RNA-seq reads carrying reference and alternative alleles over heterozygous sites and combining read count information from heterozygous loci within a gene to determine the ASE extent^6,7^. The major problem with these RNA-seq-only techniques is that the allele frequency of heterozygous variant sites in a specific chromosome may be skewed due to genetic imbalance, leading to errors if the genetic background information is missing. This is particularly important for ASE genes identified in aneuploid cancers, where the statistical approaches could fail to resolve the unbalanced heterozygous genotypes present in these samples. To measure accurately CNV states and to study ASE in the context of cancer, we developed a computational tool called Biomedswe Allele-Specific Expression-analyzer (BASE), a method consisting of functionalities to estimate the allele-specific copy number (asCN) states and to identify ASE genes by combining analysis of asCN and RNA-seq data. Specifically, our approach can detect ASE genes in aneuploid cancer samples, irrespective of the availability of whole genome sequencing (WGS) data from matched normal samples. Furthermore, we present a case study where BASE was employed to screen ASE genes using published data on high hyperdiploid acute lymphoblastic leukemia (HeH ALL), one of the most common childhood malignancies, which is characterized by gains of chromosomes X, 4, 6, 10, 14, 17, 18, and 21 in over 80% of cases^8^.

## Methods

### WGS data analysis and variants annotation in BASE

**Fig. 1:**
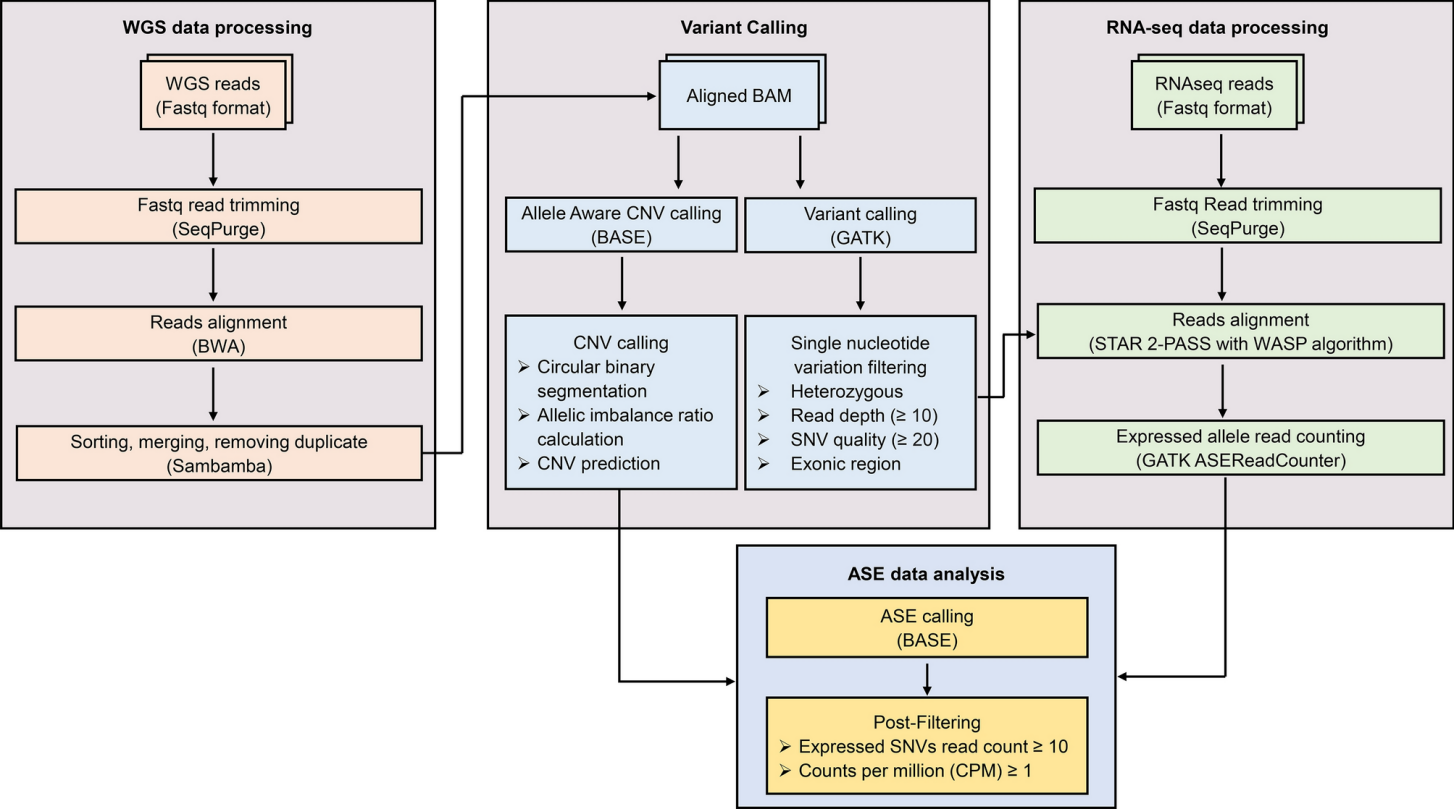
The BASE workflow is based on whole genome sequencing (WGS) and RNA sequencing (RNA-seq) paired-end fastq files from the same sample. SeqPurge filters the raw sequencing reads, whereas BWA and STAR are used for read alignment. GATK is utilized for single nucleotide variants (SNVs) calling and for determining expressed heterozygous SNV read counts. BASE performs copy number variations (CNV) calling. Finally, ASE genes are identified by integrating heterozygous SNVs read counts and copy number states.

The BASE pipeline is designed to generate a gene-based ASE analysis using matched WGS and RNA-seq data generated by next-generation sequencing (NGS) technologies (https://github.com/biomedswe/BASE). The pipeline is structured so that WGS and RNA-seq data are analyzed independently (Fig. 1). SeqPurge is used to trim the adapter sequences and the low-quality bases from raw Illumina paired-end sequencing reads^9^. The resultant trimmed WGS reads are aligned to the human genome reference build hg38 (https://hgdownload.soe.ucsc.edu/goldenPath/hg38/bigZips/analysisSet/) using BWA^10^ with sambamba^11^ used for sorting, marking duplicate reads, and indexing of alignment files. Single nucleotide variants (SNVs) are called by GATK HaplotypeCaller^12^ and only heterozygous SNVs meeting specific criteria (DP > 15, QD > 2, MQ > 35, MQRankSum > -12.5, ReadPosRankSum > -8, and FS < 60) within exonic regions of the APPRIS principal isoforms of protein-coding genes are retained. Annotation of SNVs is performed by SnpEff^13^.

### Reference panel for copy number analysis

BASE utilizes a copy number analysis reference panel compiled from unphased WGS data from 196 in-house normal samples, obtained from the population in Southern Sweden, and comprising 104 males and 92 females. This panel encompasses normalized sequencing depth data spanning around 3.1 million SNV sites across all 23 human chromosomes. For each normal sample, we performed WGS read alignment and bam file processing and called the sequencing depth of defined loci by the GATK HaplotypeCaller gvcf model^12^. We kept only SNVs with sequencing coverage over 10 and normalized the sequencing-depth using the average sequencing depth of each sample. We multiplied the normalized coverage by 2 for the X and Y chromosomes, respectively, in male samples and calculated the median value of normalized coverage across all samples for each SNV site.

### CNV calling

For WGS data from the input sample, the reference and alternative allele read depths of SNV loci present in the built-in reference panel is called using the GATK HaplotypeCaller gvcf model and only informative sites with sequencing coverage over 10 are retained. Subsequently, read counts of SNV loci are normalized to the average sequencing depth. The Log R ratio (LRR) for each SNV locus is calculated as the log-odds ratio of the sequencing depth from the investigated sample over that in the normal reference panel. The circular binary segmentation algorithm in DNAcopy^14^, using LRR data as inputs, is then employed for copy number calling. The resulting copy number levels are categorized into five classes: hemizygous deletions (1 copy), wild type (2 copies), 3 copies, 4 copies, and high amplification (≥ 5 copies).

Next, we calculate the segment allelic imbalance ratio (AI_seg_) for segments exceeding 100 kb. Briefly, for a given segment containing at least 20 heterozygous SNPs, we define the reference allele frequency (RAF) of each SNP locus as the reference allele count versus total sequencing depth in the investigated sample and calculate the AI value (AI_snp_) for each site by

$$AI_{snp} = \, abs\left( {RAF - 0.5} \right)$$

Subsequently, the unsupervised k-means clustering algorithm with a squared Euclidean distance measure is applied to all AI_snp_ values within each segment. This process divides heterozygous SNPs into two groups, and we define the mean AI_snp_ value of each group as AF_h_ and AF_L_, representing respectively the higher and lower mean AI_snp_ values of the given segment. Finally, AI_seg_ is calculated as

$$AI_{seg} = \, AF_{h} /AF_{L}$$

and the asCN states are estimated by combining the copy number levels and AI_seg_.

### RNA-seq data analysis and ASE

The trimmed RNA-seq reads are aligned to the human genome reference build hg38 (https://hgdownload.soe.ucsc.edu/goldenPath/hg38/bigZips/analysisSet/) using STAR 2-pass mapping pipeline with WASP parameters with sambamba used for bam file processing^15,16^. Only reads with mapping quality ≥ 20 and passed WASP filtering are kept. Reference and alternative allele read counts of expressed heterozygous SNVs are obtained using the GATK ASEReadCounter^12^. Heterozygous SNVs are excluded if the total read count of SNV sites is < 10. For genes within unbalanced chromosome segments, RNA-seq read counts are aggregated based on allele frequencies derived from matched WGS data for the corresponding disomic genes within the same chromosome segments. For the latter genes, we implement a pseudo-phasing procedure originally applied by Mayba et al.,^6^ where the major and minor haplotypes are assigned by alleles with higher/lower RNA-seq read counts, respectively. The binomial test is subsequently applied to examine whether the allele ratio in the RNA-seq data significantly deviates from two null hypothesis models; model 1: ratio 1 (both alleles are equally expressed, assuming the sample exhibits biallelic copy number equivalence derived from both maternal and paternal genomes) and model 2: the alleles are not equally expressed, but the expression ratios align with the allele-specific copy numbers derived from genome-wide assessments. Only genes that display ASE under the first model but not the second are defined as ASE genes driven by somatic CNVs. A false discovery rate of 0.05 and an additional threshold of allelic imbalance ratio between WGS and RNA-seq (> 2 or < 0.5) data are considered for the identification of genes associated with ASE. For gene expression quantification, the RSEM^17^ algorithm is applied using GENCODE v43 (https://www.gencodegenes.org/human/release_43.html) as the reference and only genes with CPM (counts per million) ≥ 1 are kept. To evaluate the performance of BASE in ASE calling, we compared it against two established ASE calling programs: MBASED and aScan^6,7^. For MBASED, we adhered to the guidelines from the original paper, applying an estimated MAF cutoff of ≥ 0.7 and an adjusted P-value threshold of ≤ 0.05. In the case of aSCAN, we considered only informative genes with more than 10 covered reads and used an adjusted P-value threshold of ≤ 0.05 to identify ASE genes.

### Performance and resource usage

The pipeline uses GNU parallel as the parallelization mechanism to optimize runtime^18^. All genuine tests were conducted on a computer running Linux CentOS 7 with 20 physical cores and 192 GB of RAM. The pipeline peak memory usage was ∼80 Gb for WGS data alignment using BWA. The runtime varies depending on multiple factors, such as sequencing depth and variants number of samples.

## Results

### asCN calling evaluation

By using a built-in copy number analysis reference panel, BASE provides a solution to estimate asCN changes using WGS data from tumor samples. To evaluate the precision of BASE in asCN calling, we first collected WGS data from fifteen extensively studied cancer cell lines (Supplementary Table 1) and compared the asCN calls made by BASE with those derived from matched SNP arrays, performed by the Cancer Cell Line Encyclopedia (CCLE)^19^. On average, BASE detected asCN changes with a recall rate of 98–100% in the eleven cell lines with ploidy levels ranging from 1.6 to 2.7 (Table 1). For the remaining four cell lines, exhibiting ploidy levels from 2.8 to 3.3, the BASE asCN calling result initially exhibited a low overall agreement (< 5%) with CCLE calls using default settings. However, after adjusting the tumor ploidy parameter in BASE, the recall rate was restored to > 98% (Table 1).

**Table 1:** Copy number calling results of 15 cancer cell lines and three simulated COLO 829 cancer cell lines with different tumor cell contents.

WGS dataset	Sample name	Ploidy	WGS coverage	Informative region (Mb)	Recall (Mb)	Overall accuracy (%)
Cell line WGS						
	A2058	2.21	33.10	2757.71	2740.17	99.36
	COLO 829 T	3.22	37.84	2768.17	2743.39	99.11
	HEPG2	2.26	42.04	2783.24	2776.96	99.77
	HT115	2.10	35.73	2759.91	2742.70	99.38
	KNS81	1.96	45.91	2761.61	2729.79	98.85
	LAMA84	3.11	42.52	2788.78	2777.25	99.59
	LOVO	2.20	37.91	2760.97	2749.19	99.57
	LS513	2.28	31.91	2758.90	2749.62	99.66
	MKN74	2.30	42.41	2712.25	2669.85	98.44
	MM1S	1.97	42.91	2759.18	2756.67	99.91
	MONO-MAC-1	2.09	38.35	2762.58	2744.30	99.34
	NCIH69	1.64	40.17	2717.08	2700.69	99.40
	OVCAR4	2.99	39.84	2728.81	2685.77	98.42
	SW1990	2.68	34.77	2745.19	2735.80	99.66
	T47D	2.89	40.32	2766.71	2721.87	98.38
Simulated cell line with normal cell contamination						
	COLO 829 T 75%	N.A	96.42	2754.77	2651.55	96.25
	COLO 829 T 50%	N.A	95.87	2747.81	2455.02	89.34
	COLO 829 T 25%	N.A	91.49	2779.28	1726.19	62.11

*WGS,* whole genome sequencing. *N.A.* not applicable

Tumor samples never contain 100% tumor cells but also normal cells, *e.g.*, infiltrating immune cells. This poses a problem for the accurate detection of asCN, as the reads from the tumor containing copy number aberrations are diluted with the reads from the normal diploid cells. To evaluate BASE’s asCN calling performance in samples with normal cell contamination, we obtained WGS data from the COLO 829 cell line and modelled normal cell contamination by mixing WGS reads from COLO 829R, a B lymphoblast cell line derived from the same patient. In this way, samples containing 75%, 50%, and 25% tumor cell content were modeled. For samples with a 75% tumor purity, BASE detected most asCN events with a calling accuracy rate of 96%. However, it is worth noting that chromosomal regions with loss of heterozygosity (LOH) in COLO 829 were not called correctly in the simulated samples with less than 50% tumor cell fraction due to the increase of heterozygosity within those segments (Table 1; Supplementary Fig. 1).

### Evaluation of ASE calling based on phased and unphased SNV Information

**Fig. 2:**
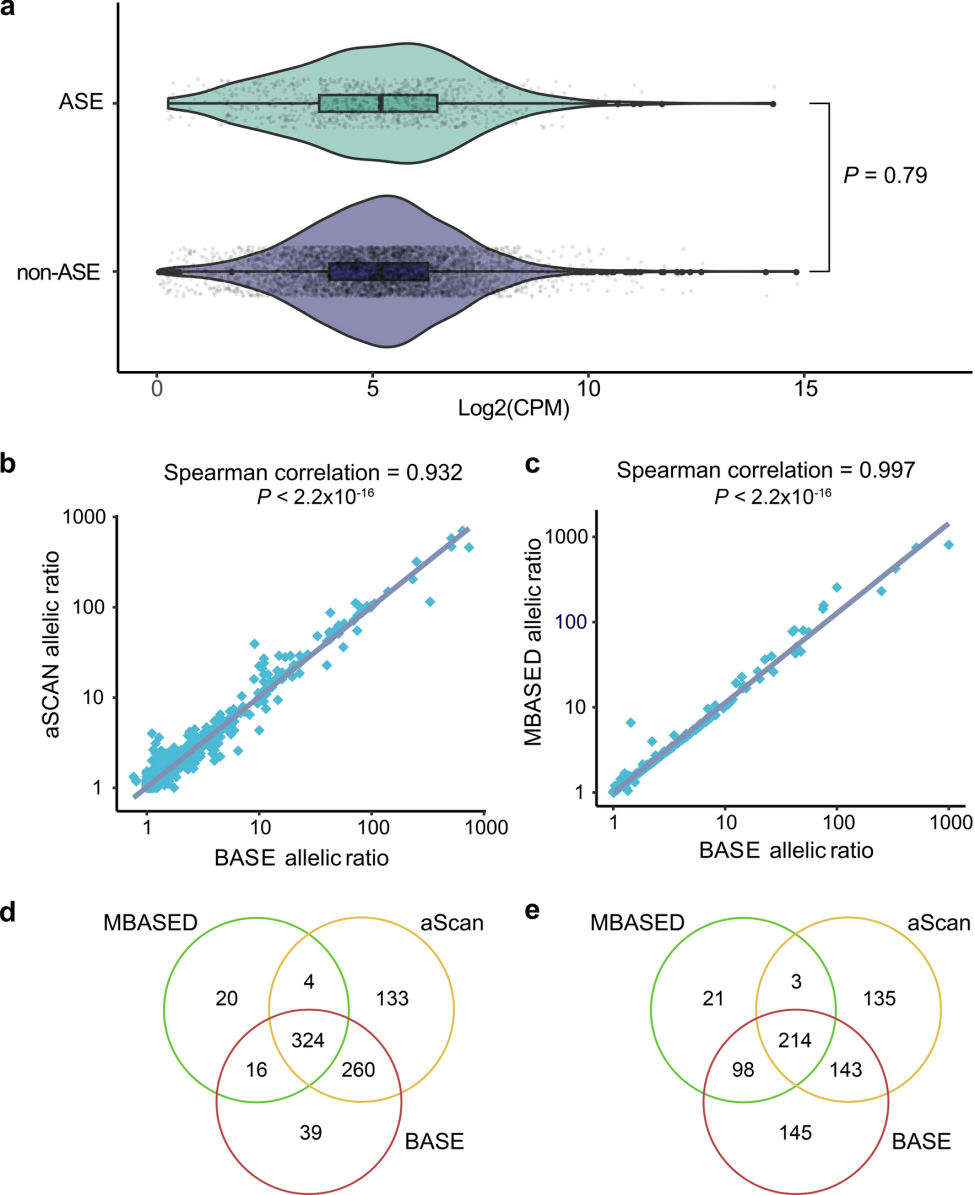
**a.** Violin plots displaying the expression of ASE genes (top) and non-ASE genes (bottom). No significant differences in gene expression levels between ASE genes and non-ASE genes were observed (*P* = 0.79, Mann–Whitney two-sided test). Gene expression levels were normalized to CPM values and then log2-transformed as shown on the x-axis. The median is denoted by the center of the boxplot, while the lower and upper hinges correspond to the first and third quartiles, respectively. Whiskers represent 1.5 times the interquartile range, with data beyond this range plotted as individual points. Spearman’s rank correlation between the allelic expression ratio derived from aSCAN and BASE (**b**), and from MBASED and BASE (**c**), respectively. The Venn diagram displays the overlap of ASE genes identified using phased (**d**) and unphased (**e**) genotype data by three tools: MBASED, aScan and BASE, respectively.

In the evaluation of BASE’s precision in identifying allelic expression events, we analyzed three replicates of HepG2 cell line RNA-seq data, where heterozygous SNPs data had been obtained from using either a phasing or non-phasing WGS analysis pipeline^20^. By using the BASE, we identified 4,933 informative expressed genes shared by the ASE calling results using either phased or un-phased genomic data as input. Of these, 639 and 600 ASE genes, respectively (Table 2), were identified, with 599 ASE genes being consistently detected in both calling results (Supplementary Fig. 2); this suggests substantial equivalence between the ASE calling results based on unphased and phased data. The relationship between the ASE state and gene expression level was investigated, revealing no gene expression profile difference between ASE and non-ASE genes, indicating that our pipeline can detect ASE regardless of the gene expression level (Fig. 2a). To further BASE’s performance in ASE calling, we compared its results with those from MBASED and aSCAN, revealing a strong correlation between the methods (Fig. 2b,c). MBASED identified 5,497 informative genes, with 364 and 336 exhibiting ASE under phased and unphased conditions, respectively. aSCAN detected 5,546 informative genes, with ASE identified in 721 genes using phased data and 495 with unphased data (Table 2). Our analysis showed that over 93% of ASE genes identified by BASE were confirmed by MBASED or aSCAN with phased data, and over 75% with unphased data (Fig. 2d,e). These findings demonstrate that BASE’s ASE calling is highly consistent with the published methods. Notably, ASE detection was slightly more accurate when based on phased SNV data compared to unphased data across all three programs, underscoring the potential benefits of using phased genetic information to enhance the precision and reliability of ASE gene detection.

**Table 2:** Overview of ASE Gene calling results across three programs in cell lines.

Cell lines	BASE	aScan	MBASED	ASE found in all three programs	ASE found in both BASE and either aSCAN or MBASED
HepG2 (phased SNVs)	639	721	364	324	600
HepG2 (Unphased SNVs)	600	495	336	214	455
NCIH2122	1710	2557	1191	1086	1658
KNS62	1044	1516	721	617	1474
SW900	1362	2075	956	869	1328

A similar approach was applied to three lung cancer cell lines, KNS62, NCIH2122, and SW900, with matched WGS and RNA-seq data derived from the CCLE (Supplementary Table 1). Our results show that the BASE program identifies over 59% of ASE genes detected across all programs, with more than 95% of the ASE genes called by BASE also recognized by at least one of the other published methods (Table 2). This suggests that BASE is robust in ASE gene calling across different tumor types.

### Application: ASE in HeH ALL

**Fig. 3:**
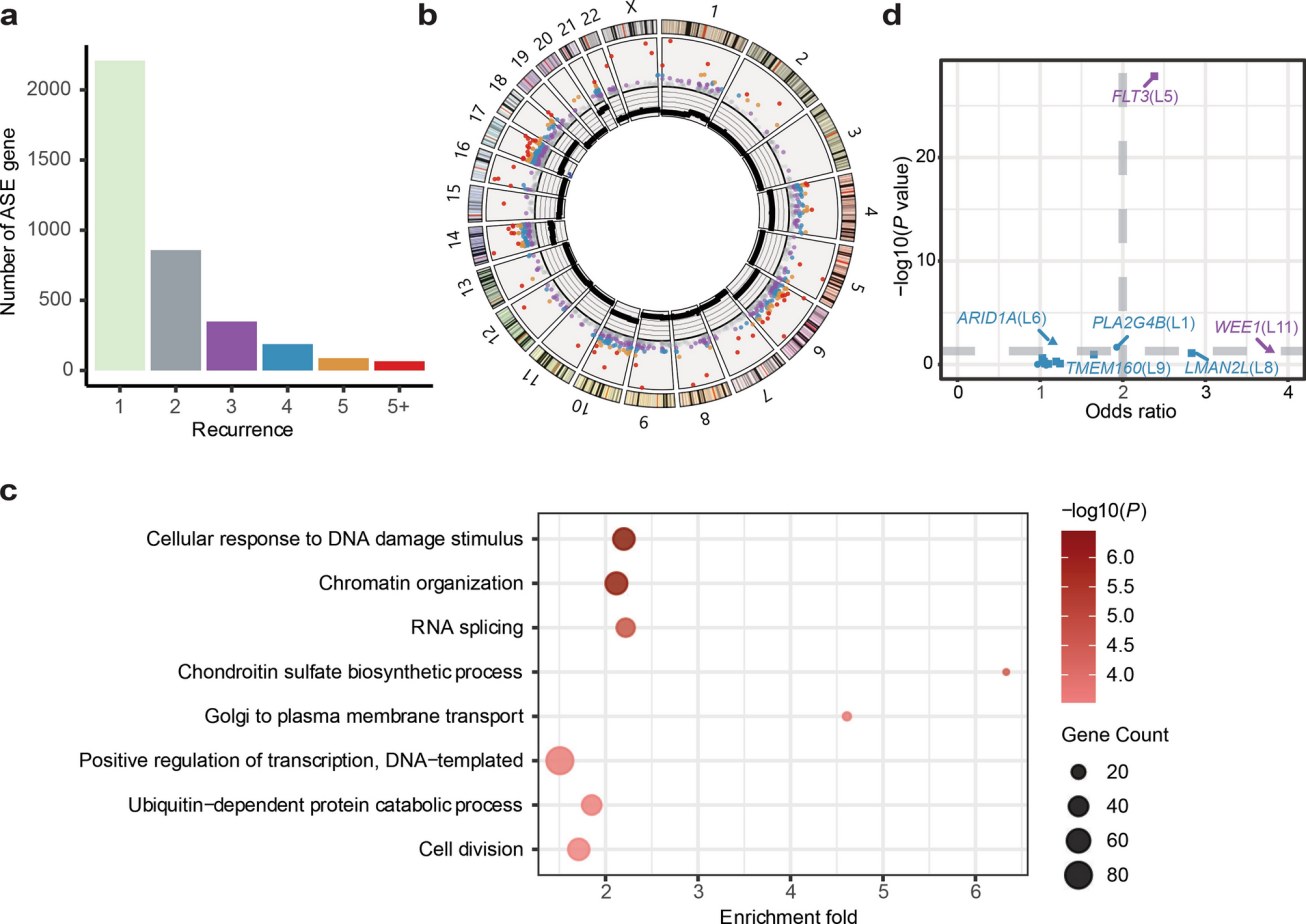
**a.** Recurrence of ASE genes across 12 HeH ALL samples. **b.** The Circos plot shows ASE genes and copy number alterations of HeH ALL samples. From outer to inner circle: chromosome numbers, chromosome ideograms, scatter plot of recurrent ASEs genes across 12 HeH ALL samples (grey, recurrence = 2; purple, recurrence = 3; blue, recurrence = 4; orange, recurrence = 5; red, recurrence > 5), and average copy number of a particular segment of 12 HeH ALL samples. **c.** Gene Ontology analysis of 1,549 recurrent ASE genes in 12 HeH ALL samples. **d.** ASE states of mutated genes in HeH ALL. ASE genes are marked in purple. The circle, square and triangle indicate the missense, nonsense and silent mutations, respectively. The x-axis indicates the odds ratio of aggregated allelic ratios from RNA-seq compared to those from WGS. The y-axis indicates the log10 transformed binomial test *P* value.

To explore the impact of CNVs on allelic expression imbalance in aneuploid tumors, we applied the BASE framework to a previously generated HeH ALL dataset (Supplementary Table 2)^5,8,21^. This dataset comprised 12 samples with matched diagnosis/remission WGS data, along with RNA-seq data from the diagnostic samples. The selection of these 12 samples was based on the criteria that all samples were sequenced using the Illumina system, ensuring consistency in sequencing technology. Additionally, to minimize batch effects and enhance comparability, all selected samples had RNA-seq data generated from sequencing libraries constructed using the same kit. Details of sample characteristics and NGS library preparations are described in the original articles. To examine ASE genes in HeH ALL, we considered only expressed genes (CPM ≥ 1) that had at least one heterozygous site covered by 10 or more RNA-seq reads. In total, we identified 8,210 expressed genes in the twelve HeH ALL samples, with 3,653 (44.5%) genes showing ASE. The majority of genes exhibited ASE in only one sample (2,372/3,653; 64.9%; Fig. 3a; Supplementary Table 3). No significant difference in the percentage of ASE genes was found between informative samples with < 80% and ≥ 80% blast cells (*P* = 0.143, two-tailed unpaired Mann–Whitney U-test; Supplementary Table 2). Additionally, we found 1,281 genes that displayed ASE in at least two HeH ALL cases, *i.e.*, were recurrent (Fig. 3b; Supplementary Table 3). Gene ontology analysis of these recurrent ASE genes revealed enrichment of the biological processes cellular response to DNA damage stimulus (GO: 0006974; *P* = 3.5 × 10^−7^), chromatin organization (GO: 0006325, *P* = 6.3 × 10^−7^), and RNA splicing (GO: 0008380, *P* = 1.7 × 10^−5^) (Fig. 3c).

### Proportion of ASE genes on trisomic versus disomic chromosomes

To investigate the relationship between non-random chromosomal gains and ASE in HeH ALL, we restricted our analysis to the 4,089 informative genes located within gained chromosomes (trisomy, tetrasomy with a 3:1 allele ratio, and pentasomy). We adapted the BASE framework to identify ASE genes that were associated with the chromosomal gains using binomial exact test under the null hypothesis model 1) each allele is equally expressed; or the alternative hypothesis model 2) two alleles are unequally expressed with the odds ratio based on asCN analysis result (Supplementary Fig. 3). Approximately 1,574 ASE genes were significantly correlated with gained chromosomes, with 22.8% (932/4,089) exhibiting recurrent ASE in at least two samples. Chromosomes 4, 6, 10, 14, 17, 18, and 21 were gained in more than 70% of HeH ALL samples and over 30% of the informative genes on these chromosomes showed allelic expression imbalance (Fig. 3b, Supplementary Table 4 and 5), indicating that the genomic imbalance caused by the copy number gains lead to widely unequal transcription of the two gene alleles in HeH ALL.

We have previously shown that genes in the gained chromosomes X, 14, and 21 are associated with the strongest dosage effects and that gains of chromosome 21 had the highest selective advantage in HeH ALL^5,8^. In line with this, we observed a higher frequency of ASE in chromosomes 14 and 21 (over 48%) in contrast to the other commonly gained chromosomes (range 38.4% to 45.9%), indicating that allelic imbalance in the expression of genes on these two chromosomes may be a key hallmark in HeH ALL (Supplementary Table 5). In contrast, when examining informative genes outside of the gained chromosomes, only 4.6% (294/6,441) showed recurrent ASE, highlighting the predominant influence of copy number gain events on ASE in HeH ALL (Fig. 3b, Supplementary Table 3).

To explore further whether the leukemia-related ASE events could be driven by chromosomal gains, we investigated ASE events involving known cancer driver genes reported in OncoKB (https://www.oncokb.org/cancer-genes). A total of 54 oncogenes, including four that are known to be mutated in leukemia (*NSD2*, *CCND3*, *U2AF1*, and *JAK2*)^22^, showed ASE in 7/12 HeH ALL samples in trisomic chromosomes, suggesting that the expression of a set of well-known leukemia-related genes could also be altered by gene dosage in HeH ALL, although other causes of ASE cannot be excluded. However, only a few leukemia-related ASE events were observed in disomic chromosomes. For example, *KMT2C* is frequently mutated in multiple human cancers and heterozygous germline mutations in *KMT2C* have been reported to be enriched in infants with leukemia^23^. Here we observed ASE of *KMT2C* in three samples with disomy 7, indicating that the selective expression of specific alleles of *KMT2C* may play a role in the development and progression of HeH ALL.

### ASE associated with somatic mutations

We next examined ASE involving mutated genes in HeH ALL by analyzing somatic SNVs from paired tumor/normal WGS data. Only coding region mutations (annotated as “silent” “missense” or “nonsense”) were included. We observed 14 somatic mutation sites that were expressed across 12 genes. Among these, the genes *FLT3* and *WEE1* showed ASE in two HeH cases (Fig. 3d). *FLT3* overexpression is a known phenomenon in several leukemias, such as HeH ALL, irrespective of mutational status^24^. In this study, we found the HeH sample L5 that had a hemizygous deletion near the *FLT3* promoter region, displayed cis-dysregulation of *FLT3* expression and increased expression of the mutated *FLT3* allele as previously reported^25^. Overexpression of *WEE1*, a nuclear kinase that is involved in cell cycle G2-M checkpoint signaling, is associated with a poor outcome in various hematological malignancies and inhibition of WEE1 is considered as a potential therapeutic target in AML^26^. Here we identified a heterozygous G > T mutation in HeH sample L11, resulting in a premature stop codon and triggering nonsense-mediated decay in *WEE1*. In line with this, a significant allelic expression imbalance favoring the overexpression of the wild-type allele of *WEE1* was observed. However, the functional consequence of this nonsense mutation in HeH is unknown and further investigations of larger cohorts are needed to elucidate the function of *WEE1* mutations in HeH ALL. Collectively, these results suggest that ASE can also be associated with somatic mutations in HeH, in line with previous observations in other types of tumors^2^.

## Discussion

Detecting ASE is crucial for understanding the impact of cis-regulatory elements on RNA expression in tumors. Because ASE is assessed within the same cellular context, it naturally mitigates variability caused by experimental conditions such as sample preparation and sequencing depth. Consequently, ASE serves as an outstanding tool for analyzing allelic gene expression differences that arise from somatic aberrations and for investigating the pathogenic consequences of these aberrations in cancers. However, existing methods that solely rely on RNA-seq data for identifying ASE have certain limitations. For example, not using matched genomics data, such as asCN, may lead to incorrect biological interpretations of gene expression regulation caused by CNVs, especially in the context of aneuploidy, which is common in cancer. Population-based ASE analysis is another approach to ASE calling that is often considered robust for identifying ASE genes across large cohorts^27^. However, its utility in oncology is limited due to the heterogeneous and complex nature of genetic alterations in cancers, such as mutations, recombinations, and CNVs, which often surpass the complexity found in healthy tissues or less aggressive diseases. Since many oncogenic alterations are specific to individual samples, these population-based approaches may miss ASE events that are present in only a small number of cancer samples, even when those share the same cancer type.

Here, we integrated asCN data into ASE calling algorithms. Our aim was to discern asCN-derived ASE events by comparing asCN-aware ASE calling results to allelic balanced ASE calling results. This integration helps distinguish passenger events from driver events around recurrent CNVs. Additionally, the BASE pipeline incorporates an asCN calling approach that utilizes a comprehensive copy number analysis reference panel, enabling the estimation of asCN alterations using WGS data extracted from tumor samples, regardless of the availability of matched normal samples.

Using publicly available cell line data, we demonstrated that BASE is able to detect asCN with a low rate of false calls. However, it is important to note that BASE is not optimal to identify asCN in tumor samples with high levels of noise, such as highly heterogeneous samples or tumor samples with high normal cell contamination. The asCN calling performance in regions with LOH could be compromised by the heterozygosity and allelic imbalance ratio changes within those regions. Similar problems are commonly encountered in various CNV calling tools, and specialized methods are required to call CNVs in complex tumor samples^28^.

Previous studies have shown that integrating phased variants from long-read sequencing and RNA-seq read counts can enhance the power to detect ASE^29,30^. However, due to the high requirements of DNA sample quality for long-read sequencing, this approach is not universally available for clinical samples. Moreover, precise haplotype phasing across entire chromosomes is challenging, especially in cancer cells with numerous CNVs. In our study, we investigated the ASE calling performance using heterozygous SNP data obtained from both unphased and phased WGS data from the HepG2 cell line. Our results indicated a similar accuracy for both types of data, underlining the robustness of the ASE calling workflow.

Furthermore, we demonstrated the credibility of ASE effects uncovered by BASE through the analysis of paired WGS and RNA-seq datasets from HeH ALL, one of the most common childhood malignancies, which generally shows a similar pattern of chromosomal gains in the majority of samples^8^. This makes HeH ALL a valuable model system for studying the effects of aneuploidy on ASE. Our analysis identified a general trend of ASE in relation to chromosomal gains in HeH ALL, with a significant number of genes on gained chromosomes exhibiting recurrent ASE events. Notably, chromosomes 14 and 21 showed stronger effects on ASE compared to other commonly gained chromosomes. This agrees well with previous findings of these two chromosomes being associated with particularly strong dosage effects in ALL, since the latter would be expected to arise from the homologue that is present in two copies in trisomic chromosomes, leading to ASE, and underscores their importance in the pathogenesis of HeH ALL^5,8^.

In HeH ALL, non-CNV derived recurrent ASE events are rare. In some samples, ASE in the absence of a CNV can be attributed to mutations causing leukemia. For instance, our analysis observed the overexpression of a mutated *FLT3* allele in one HeH ALL through the recently described enhancer mechanism^25^. Furthermore, besides the well-known leukemia-related genes, our analysis also identified novel potential driver mutations, such as *WEE1*, associated with selective expression of mutated alleles in HeH ALL. However, given the small number of samples in this study, further investigations involving larger cohorts of HeH ALL are essential for determining novel tumor suppressor and oncogenes.

In summary, we have developed BASE, an automated ASE analysis framework employing combined genome and transcriptome sequencing data from human samples. BASE provides an asCN calling algorithm applying NGS data from tumor-only samples for which no normal samples are available by taking advantage of a built-in reference panel for CNV analysis. By integrating the asCN data into ASE calling algorithms, BASE provides a simple and effective way to interpret the molecular mechanisms underlying ASE and makes it possible to identify driver events that are caused by CNVs in cancer.

## Supplementary Information

Supplementary Information 1.

Supplementary Information 2.

## Data Availability

The present study analyzes existing, publicly available data. The Cancer Cell Line Encyclopedia (CCLE) next generation sequencing data of 18 extensively studied cancer cell lines used in this study were obtained from the NCBI Sequence Read Archive (SRA; https://www.ncbi.nlm.nih.gov/sra) under accession number SRP186687. The WGS data generated of the HepG2 cell line are available via ENCODE (https://www.encodeproject.org/) under accession numbers ENCBS760ISV. HepG2 RNA-seq data can be accessed via ENCODE under accession numbers ENCLB707CLT and NCBI SRA under accession numbers SRR8616129 and SRR629573. WGS and RNA-seq data of the 12 HeH ALL samples analyzed in this study are available from the European Genome-phenome Archive (EGA; https://ega-archive.org/) under accession numbers EGAD00001008988 and EGAD00001002112, respectively. The code used to perform the analysis is available on Github (https://github.com/biomedswe/BASE).
