# Supplementary material for: Characterizing the allele-specific gene expression landscape in high hyperdiploid acute lymphoblastic leukemia with BASE
Source: Sci Rep. 2024 Oct 5;14:23181. doi: 10.1038/s41598-024-73743-8 (PMC11455916; doi:10.1038/s41598-024-73743-8)
Supplement: Supplementary file 1 — Supplementary Information 1. [file 41598_2024_73743_MOESM1_ESM.docx]

**Supplementary Information for**

**Characterizing the allele-specific gene expression landscape in high hyperdiploid acute lymphoblastic leukemia with BASE**

Jonas Andersson^1,2^, Efe Aydın^1^, Rebeqa Gunnarsson^1^, Henrik Lilljebjörn^1^, Thoas Fioretos^1,3^, Bertil Johansson^1,3^, Kajsa Paulsson^1^ & Minjun Yang^1*^

^1^Department of Laboratory Medicine, Division of Clinical Genetics, Lund University, Lund, Sweden. ^2^Lund University Diabetes Centre, Department of Clinical Sciences Malmö, Lund University, Malmö, Sweden. ^3^Department of Clinical Genetics, Pathology, and Molecular Diagnostics, Office for Medical Services, Laboratory Medicine, Region Skåne, Lund, Sweden.

*e-mail: [minjun.yang@med.lu.se](mailto:minjun.yang@med.lu.se)

| **Supplementary Table 1.** The cancer cell lines, information from the Cancer Cell Line Encyclopedia (CCLE), and supporting data availability |  |  |  |  |  |
| --- | --- | --- | --- | --- | --- |
| \| Cohort \| Cell line \| Disease subtype \| Lineage \| Gender \| Purity^a^ \| Experiment Accession \| Study Accession \| \| --- \| --- \| --- \| --- \| --- \| --- \| --- \| --- \| \| asCN calling evaluation \| A2058 \| Melanoma \| Skin \| Male \| 1 \| SRX5437602 \| SRP186687 \| \| HEPG2 \| Hepatoblastoma \| Liver \| Male \| 1 \| SRX5437591 \| SRP186687 \| \| HT115 \| Colon Adenocarcinoma \| Bowel \| Unknown \| 1 \| SRX5449789 \| SRP186687 \| \| KNS81 \| Glioblastoma \| CNS/Brain \| Male \| 1 \| SRX5449831 \| SRP186687 \| \| LAMA84 \| Chronic Myeloid Leukemia, BCR::ABL1+ \| Myeloid \| Female \| 0.99 \| SRX5449809 \| SRP186687 \| \| LOVO \| Colon Adenocarcinoma \| Bowel \| Male \| 1 \| SRX5449802 \| SRP186687 \| \| LS513 \| Colorectal Adenocarcinoma \| Bowel \| Male \| 1 \| SRX5449801 \| SRP186687 \| \| MKN74 \| Tubular Stomach Adenocarcinoma \| Esophagus/Stomach \| Male \| 1 \| SRX5449781 \| SRP186687 \| \| MM1S \| Plasma Cell Myeloma \| Lymphoid \| Female \| 1 \| SRX5449768 \| SRP186687 \| \| MONO-MAC-1 \| Acute Monoblastic/Monocytic Leukemia \| Myeloid \| Male \| 1 \| SRX5449769 \| SRP186687 \| \| NCIH69 \| Small Cell Lung Cancer \| Lung \| Male \| 1 \| SRX5466679 \| SRP186687 \| \| OVCAR4 \| High-Grade Serous Ovarian Cancer \| Ovary/Fallopian Tube \| Female \| 1 \| SRX5466653 \| SRP186687 \| \| SW1990 \| Pancreatic Adenocarcinoma \| Pancreas \| Male \| 0.99 \| SRX5466668 \| SRP186687 \| \| T47D \| Breast Invasive Ductal Carcinoma \| Breast \| Female \| 1 \| SRX5466706 \| SRP186687 \| \| COLO 829 tumor^a^ \| Cutaneous Melanoma \| Skin \| Male \| 1 \| ERR2752450 \| PRJEB27698 \| \| COLO 829 blood^a^ \| B lymphoblast cell line \| B-cells \| Male \| N.A. ^b^ \| ERR2752449 \| PRJEB27698 \| \| ASE calling evaluation \| KNS62 \| Non-Small Cell Lung Cancer \| Lung \| Male \| 1 \| SRX5449830^c^  SRX5414691 \| SRP186687 \| \| NCIH2122 \| Non-Small Cell Lung Cancer \| Lung \| Female \| 1 \| SRX5449842^c^  SRX5414587 \| SRP186687 \| \| SW900 \| Non-Small Cell Lung Cancer \| Lung \| Male \| 1 \| SRX5466711^c^  SRX5414547 \| SRP186687 \|   ^a^Purity refers to the fraction of tumor cell.  ^b^N.A., not applicable.  ^c^NCBI SRA accession numbers for whole genome sequencing data, followed by the accession numbers for RNA sequencing data |  |  |  |  |  |

**Supplementary Table 2**. Patient information including clinical data, supporting data availability and inclusion in previous publications.

| CaseID | Gender | Age | Bone marrow blast % | Karyotype^a,b^ | WGS reads number | RNA-seq reads number | Lilljebjörn et al. 2016 | Yang et al. 2019 | Woodward et al. 2023 |
| --- | --- | --- | --- | --- | --- | --- | --- | --- | --- |
| L1 | M | 2 | NK | 56,XY,+X,+Y,+4,t(?5;9)(q?;p13-21),+6,+10,+14,+17, +18,+21,+22 | 1 494 439 715 | 14 358 420 | case 12 | HeH_19 | L1 |
| L2 | F | 15 | NK | 57,XX,+X,+X,+4,+6,der(8)t(8;14)(p11;q12),+10,+14, +14,+17,+18,+21,+21 | 1 273 099 401 | 9 547 058 | case 27 | HeH_26 | L15 |
| L3 | M | 3 | 94 | 55,XY,+X,dup(1)(q12q25),+4,+6,+10,+14,+17,+18, +21,+21 [based on FISH and SNP array analysis] | 1 416 246 871 | 11 176 446 | case 119 | HeH_4 | L25 |
| L4 | M | 3 | 95 | 56,XY,+X,der(1)?ins(1;?)(q21;?),+4,+6,+8,+10,+14, +17,+18,+21,+21 | 1 242 890 065 | 19 322 856 |  | HeH_5 | L29 |
| L5 | M | 3 | NK | 55,XY,+X,+4,+5,+6,idic(7)(p11),+8,+10,+14,+17,+21 | 1 510 754 706 | 10 655 901 | case 56 | HeH_6 | L31 |
| L6 | F | 3 | 40 | 58,XX,+X,+4,+6,+9,+10,+11,+12,+14,+17,+18,+21,+21 | 1 450 702 064 | 8 130 183 | case 104 | HeH_34 | L32 |
| L7 | M | 1 | 90 | 55,XY,+X,+4,+6,+14,+17,+18,+21,+21,+22 | 1 554 485 225 | 5 960 830 | case 23 | HeH_37 | L38 |
| L8 | M | 3 | NK | 54,XY,+X,+4,+6,+10,+14,+17,+18,+21 | 1 517 453 154 | 20 530 708 | case 28 | HeH_7 | L39 |
| L9 | M | 13 | 90 | 55,XY,+X,t(2;8)(p11.2;q21.13),+4,+6,+10,+14,+der(17)t(17;19)(q?;?)del(17)(p11p13),+18,der(19)t(17;19), +21,+21 | 1 436 017 187 | 11 269 963 | case 175 | HeH_17 | L58 |
| L10 | M | 1 | 67 | 54,XY,+X,+6,+14,+15,+17,+18,+21,+21 | 1 517 240 009 | 13 405 021 | case 96 | HeH_1 | L60 |
| L11 | M | 6 | 62 | 57-58,XY,+X,+4,+6,+9,+10,+14,+17,+18,+18,+21,+21 | 1 478 457 769 | 10 619 917 | case 188 | HeH_45 | L65 |
| L12 | M | 4 | 80 | 56,XY,+X,+3,+4,+6,+8,+10,+14,+16,+18,+21/56,idem, i(7)(q10) | 1 476 434 206 | 12 634 902 | case 162 | HeH_12 | L72 |

^a^Karyotypes based G-banding, single nucleotide polymorphism array analysis and/or whole genome sequencing (WGS).

^b^The karyotypes of all cases have been previously published in Yang et al (2019)

Abbreviations: F, female; M, male; NK, not known.

**Supplementary Table 4**. Number of ASE genes associated with chromosome gains in high hyperdiploid acute lymphoblastic leukemia samples.

| CaseID | Informative genes with  expressed heterozygous SNP sites | ASE genes | ASE genes caused by  chromosome gains |
| --- | --- | --- | --- |
| L1 | 2,714 | 537 | 355 |
| L2 | 3,252 | 559 | 292 |
| L3 | 3,151 | 494 | 316 |
| L4 | 3,622 | 826 | 580 |
| L5 | 2,267 | 488 | 396 |
| L6 | 2,683 | 467 | 256 |
| L7 | 2,397 | 350 | 212 |
| L8 | 3,555 | 761 | 502 |
| L9 | 2,902 | 549 | 313 |
| L10 | 3,403 | 479 | 292 |
| L11 | 3,200 | 414 | 272 |
| L12 | 3,100 | 739 | 431 |

ASE, allelic-specific expression; SNP, single nucleotide polymorphism.

**Supplementary Table 5**. Summary of ASE genes associated with commonly acquired chromosomes in high hyperdiploid acute lymphoblastic leukemia samples.

| Chromosome | Informative gene | Number of cases with chromosome gains | Number of recurrent ASE genes | Percentage (%) |
| --- | --- | --- | --- | --- |
| Chr21 | 95 | 12 | 50 | 52.6 |
| Chr14 | 288 | 12 | 140 | 48.6 |
| Chr18 | 122 | 11 | 56 | 45.9 |
| Chr17 | 523 | 11 | 240 | 45.9 |
| Chr6 | 447 | 12 | 198 | 44.3 |
| Chr10 | 327 | 10 | 133 | 40.7 |
| Chr4 | 302 | 11 | 116 | 38.1 |


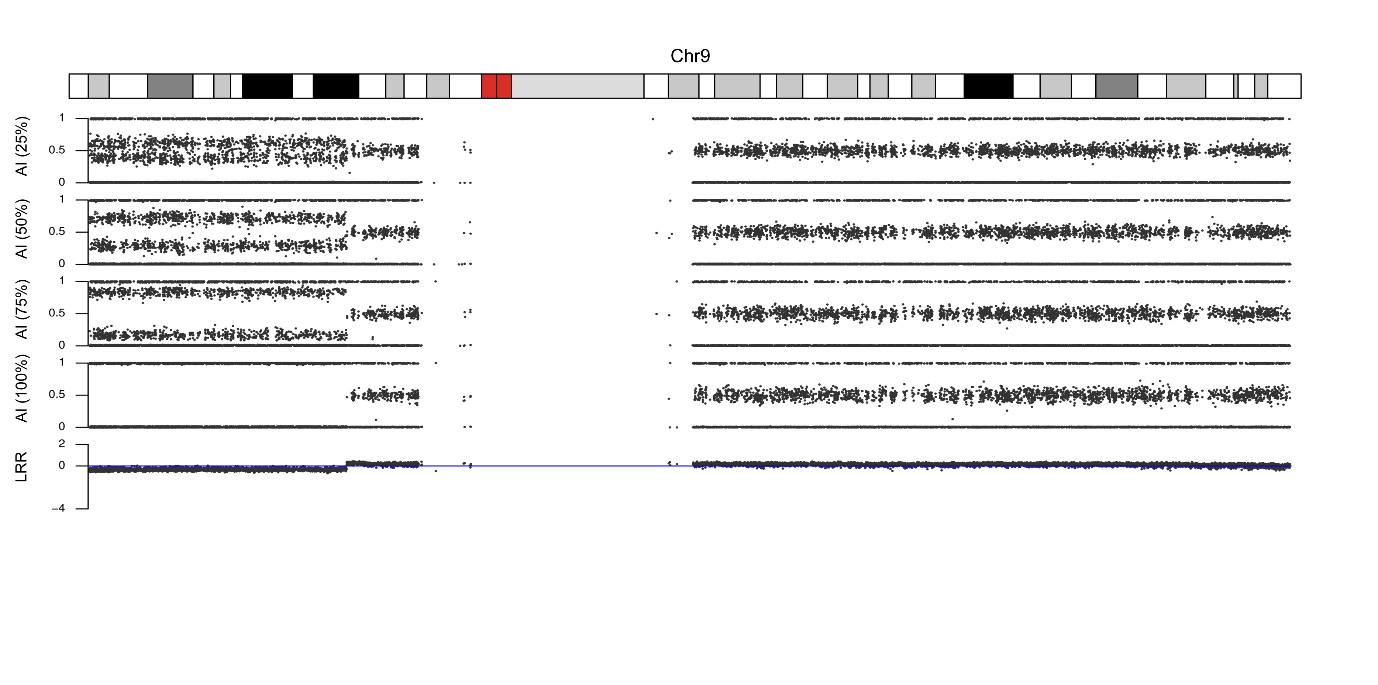


**Supplementary Figure 1.** Allelic imbalance (AI) and Log2 ratio (LRR) of chromosome 9 in cancer cell line COLO 829 and simulated cancer cell line COLO 829 with varied tumor cell content. From top to bottom: ideogram of chromosome 9, AI signal in simulated whole genome sequencing (WGS) data with 25%, 50%, 75%, and 100% of COLO 829 cancer cells; LRR of WGS data with 100% COLO 829 cancer cells, with segmentation results indicated by blue lines. The AI signal diminishes as heterozygosity increases within the chromosome arm 9p segment.


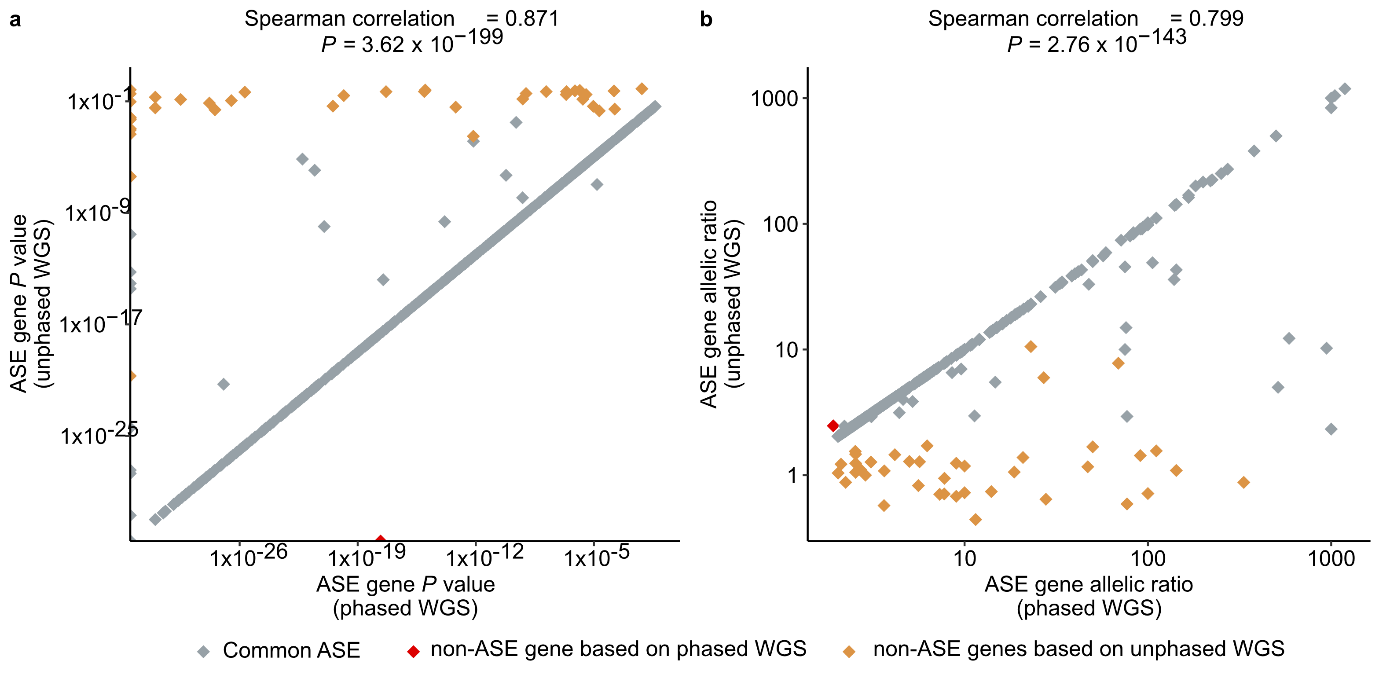


**Supplementary Figure 2.** Comparison of allele-specific expression (ASE) calling result based on phased and unphased HepG2 whole genome sequencing (WGS) data. Spearman correlation of allelic imbalance p values (**a**) and allelic imbalance ratios (**b**) based on phased and unphased HepG2 WGS data.

**Supplementary Figure 3.** The Manhattan plot illustrates the allele-specific expression (ASE) of genes in 12 high hyperdiploid acute lymphoblastic leukemia samples. The left panel shows ASE calling results using Binomial test with null hypothesis model 1, while the right panel presents results from Binomial test with null hypothesis model 2 (main text method). Gray dots represent non-ASE genes, yellow dots indicate non-ASE genes exhibiting statistically significant allelic imbalance, and red dots highlight ASE genes with both statistically significant allelic imbalance and allelic odd ratio >2 or <0.5.


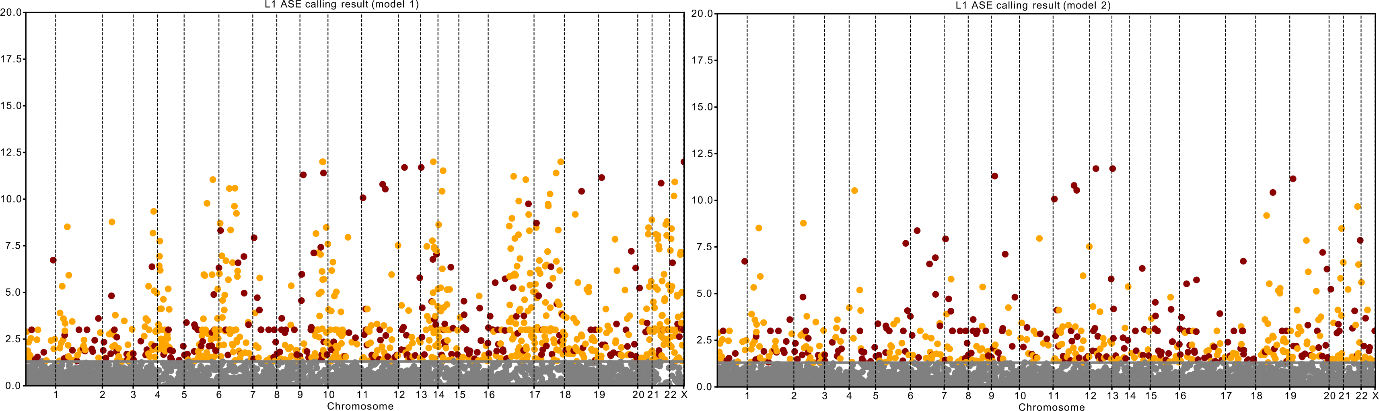


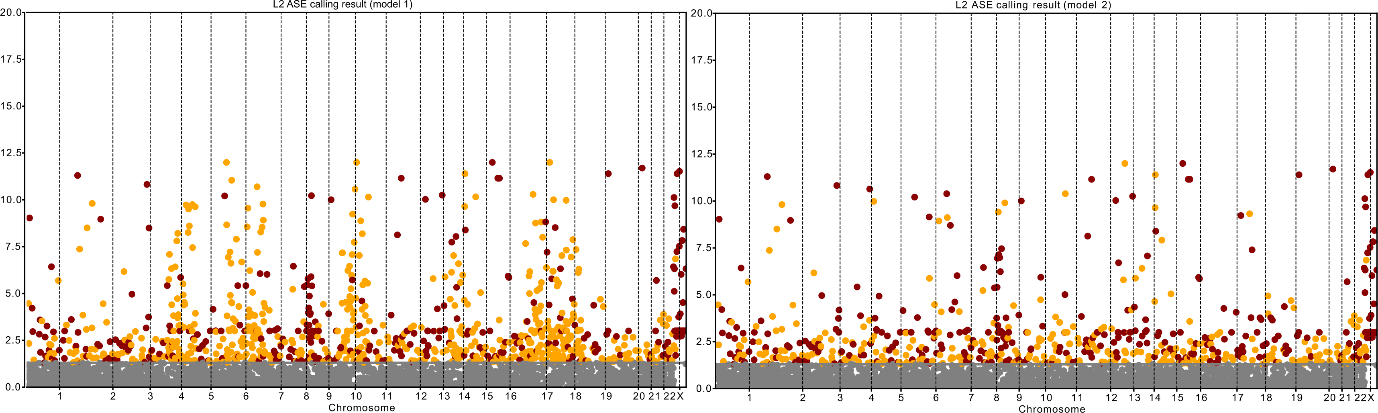


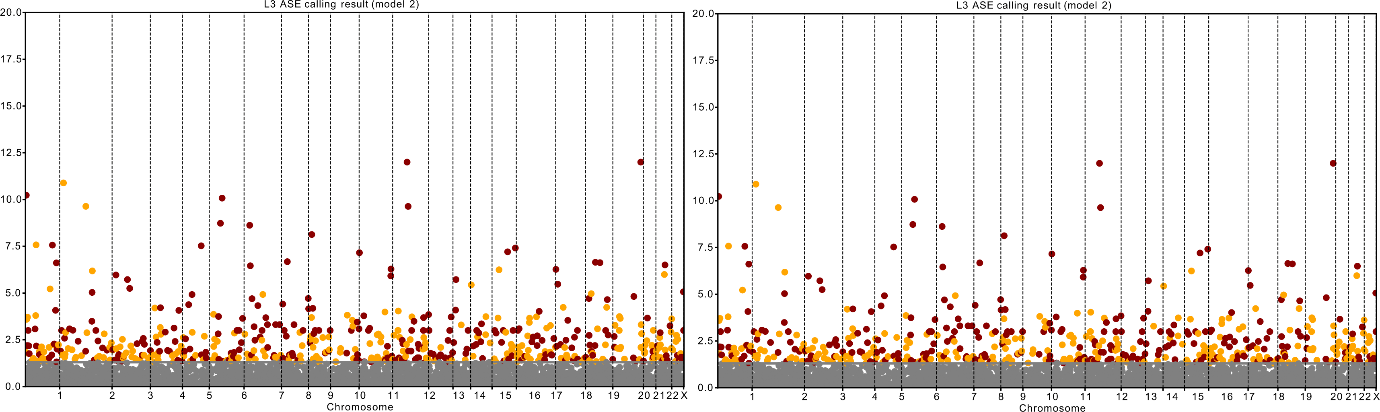


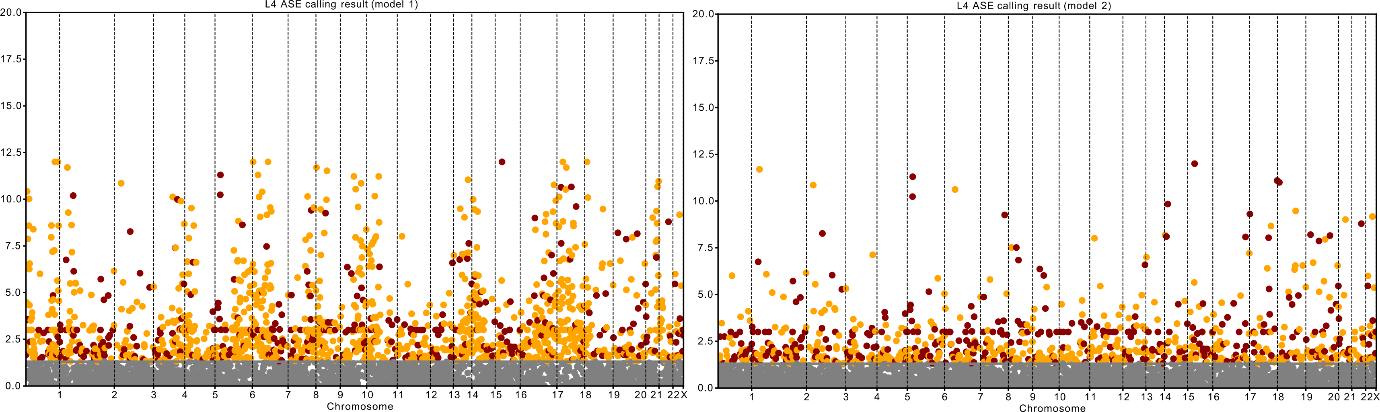


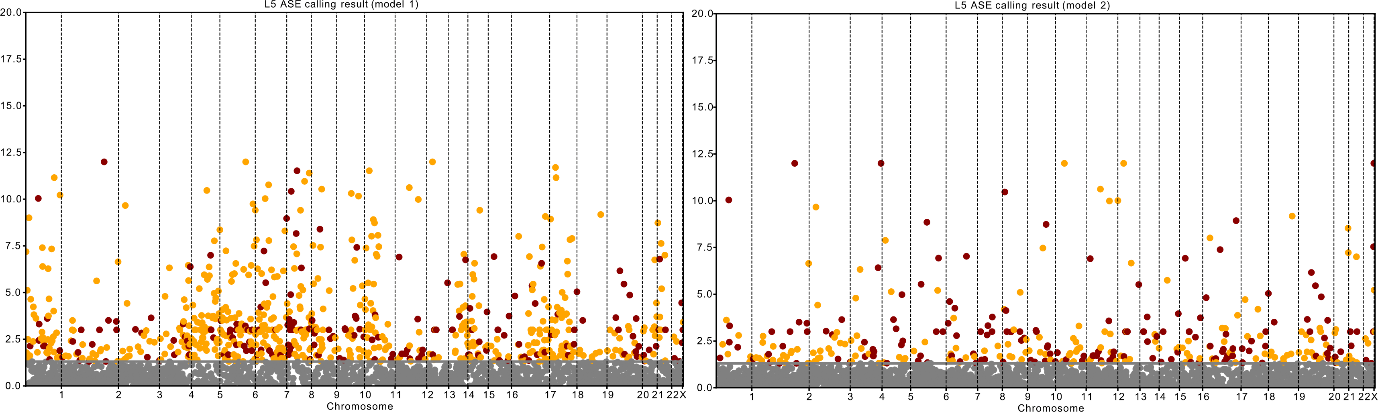


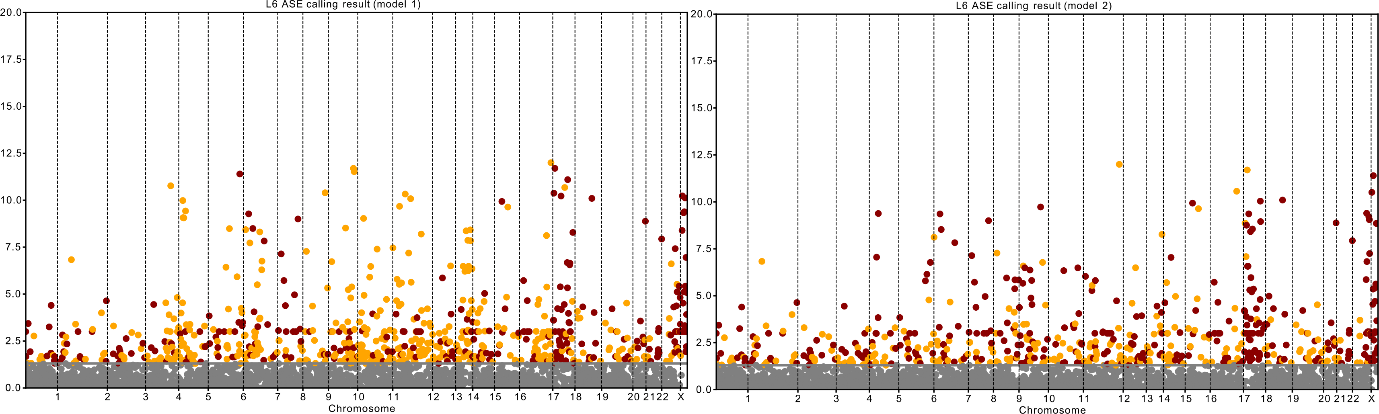


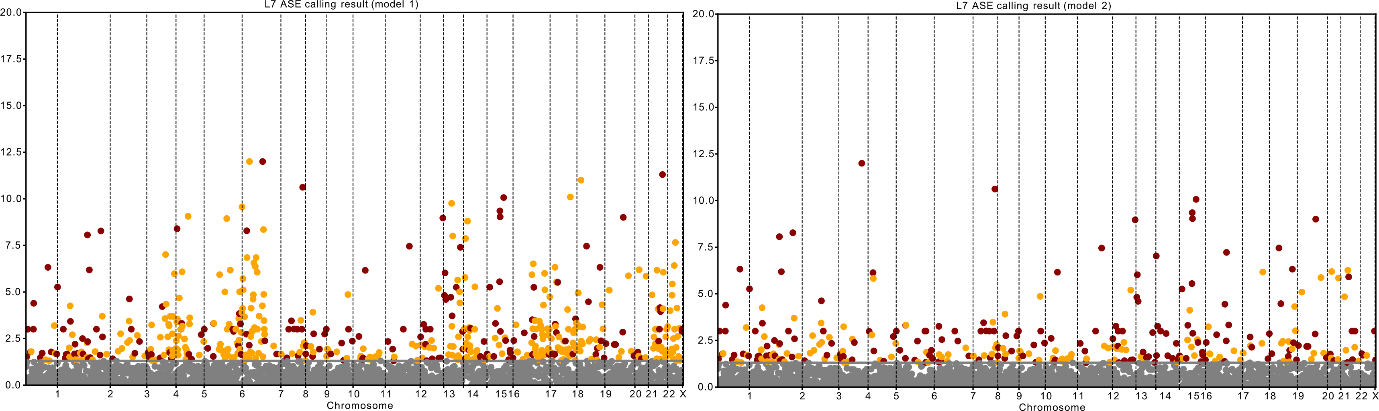


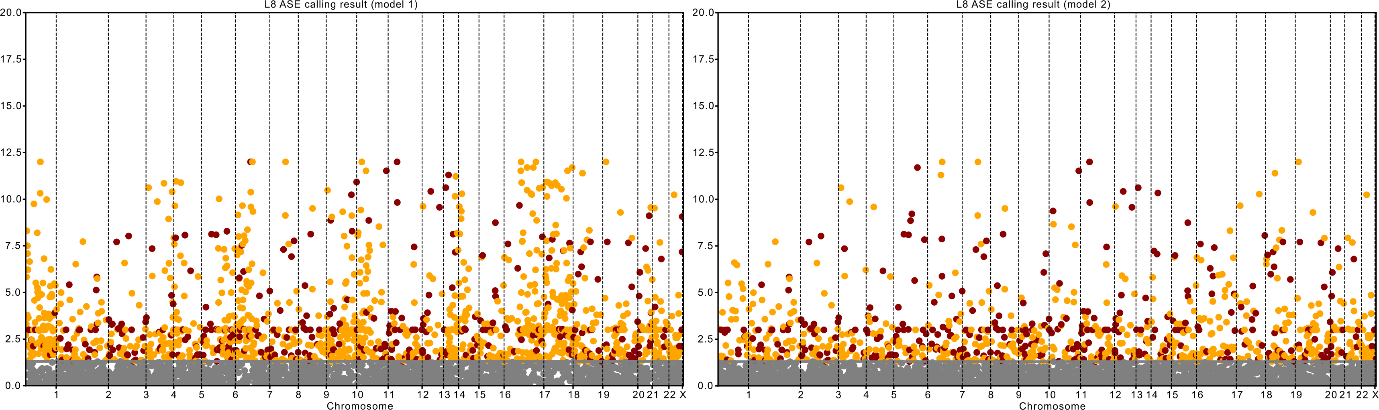


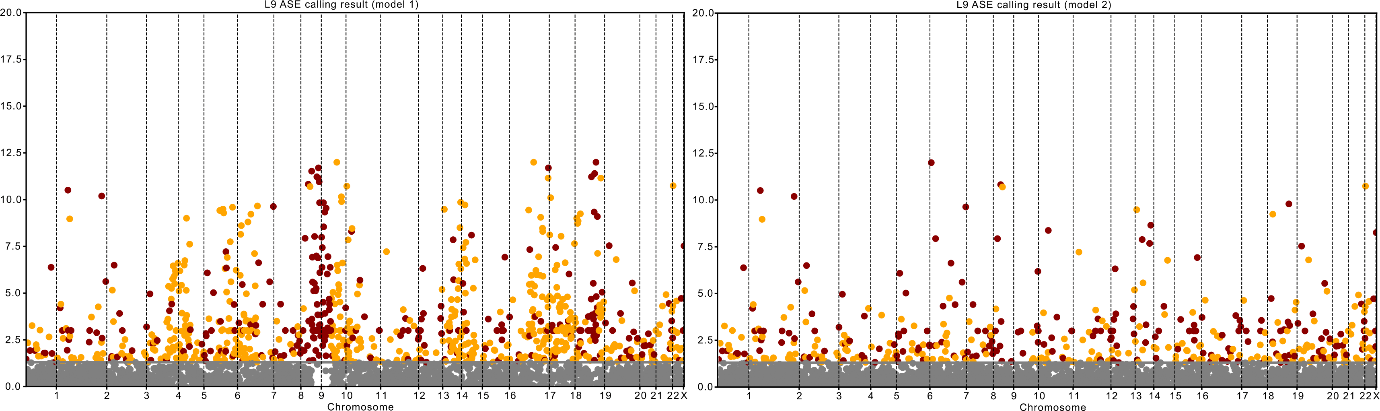


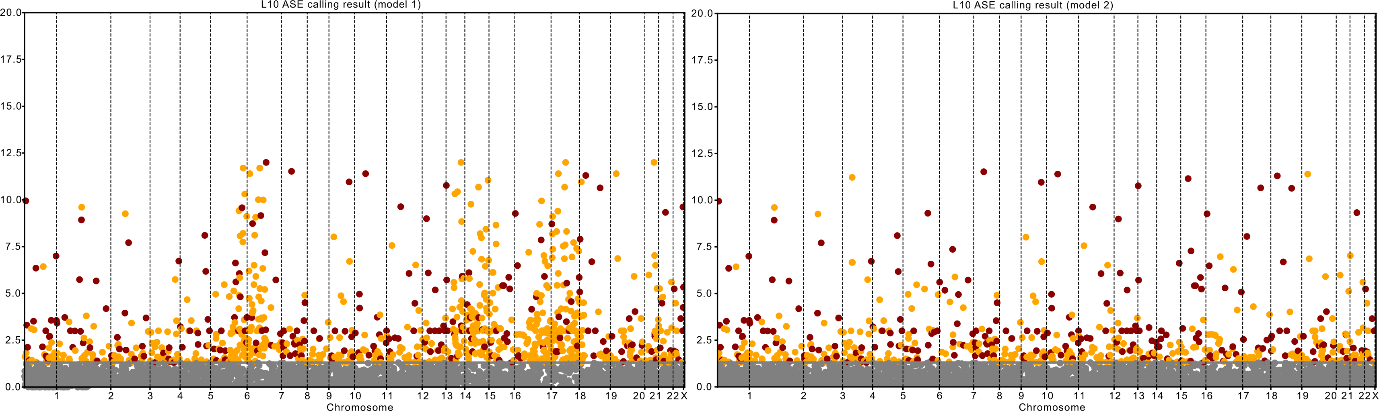


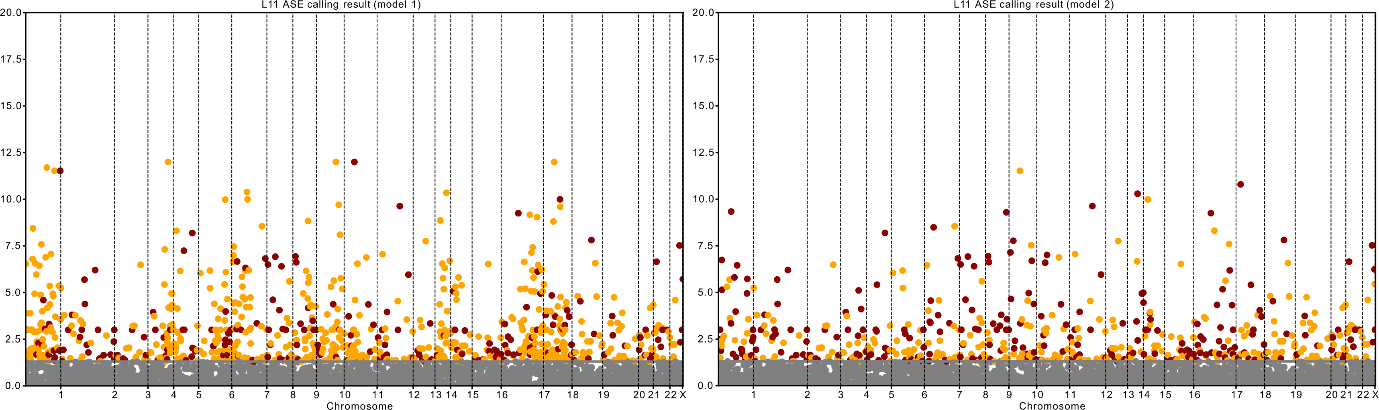


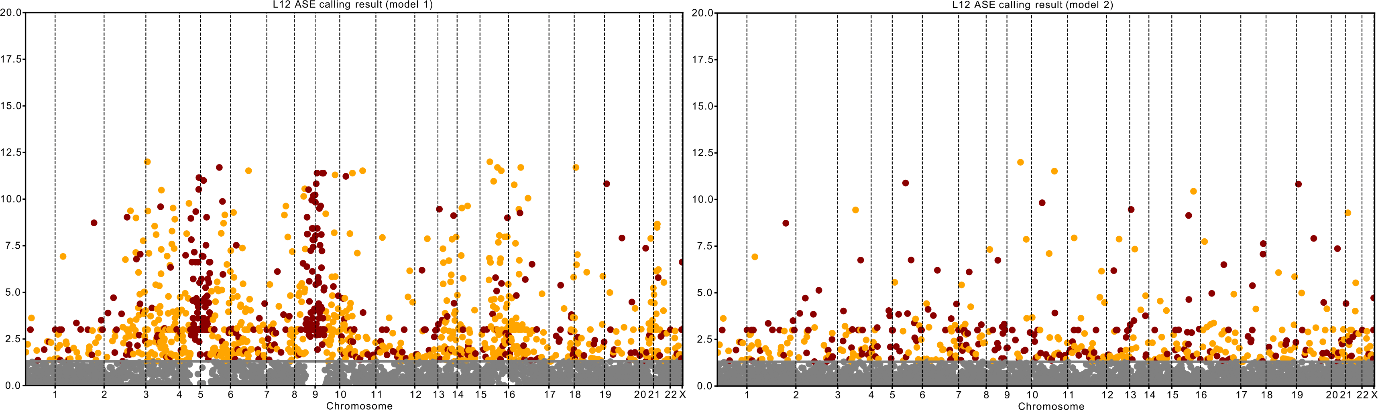


**References**

1. Lilljebjörn, H. *et al.* Identification of ETV6-RUNX1-like and DUX4-rearranged subtypes in paediatric B-cell precursor acute lymphoblastic leukaemia. *Nat Commun* **7**, 11790, doi:10.1038/ncomms11790 (2016).
2. Woodward, E. L. *et al.* Clonal origin and development of high hyperdiploidy in childhood acute lymphoblastic leukaemia. *Nat Commun* **14**, 1658, doi:10.1038/s41467-023-37356-5 (2023).
3. Yang, M. *et al.* Proteogenomics and Hi-C reveal transcriptional dysregulation in high hyperdiploid childhood acute lymphoblastic leukemia. *Nat Commun* **10**, 1519, doi:10.1038/s41467-019-09469-3 (2019).
